# Protective role of Cecropin AD against LPS-induced intestinal mucosal injury in chickens

**DOI:** 10.3389/fimmu.2023.1290182

**Published:** 2023-12-14

**Authors:** Yan Zhi, Tingyu Li, Yaxuan Li, Tao Zhang, Mengze Du, Qian Zhang, Xiangdong Wang, Ge Hu

**Affiliations:** ^1^ College of Animal Science and Technology, Beijing University of Agriculture, Beijing, China; ^2^ Department of Otolaryngology Head and Neck Surgery, Beijing TongRen Hospital, Capital Medical University, the Key Laboratory of Otolaryngology-Head and Neck Surgery (Ministry of Education of China), Beijing Key Laboratory of Nasal Diseases, Beijing Institute of Otolaryngology, Beijing, China

**Keywords:** Cecropin AD (CAD), lipopolysaccharide (LPS), intestinal mucosal injury, oxidative stress, inflammatory markers, NLRP3 inflammasome

## Abstract

**Introduction:**

Cecropin AD (CAD), a renowned antimicrobial peptide, has shown promising potential in treating various bacterial infections. This study investigates the protective effects of CAD against lipopolysaccharide (LPS)-induced intestinal adversities in chickens.

**Methods:**

Sixty SPF-grade chicks were divided into groups and exposed to different dosages of CAD, followed by LPS administration. The study assessed the impact of CAD on intestinal mucosal injury markers, oxidative stress, and inflammation.

**Results:**

LPS significantly increased Diamine oxidase (DAO) and D-lactate (D-LA) levels, both indicators of intestinal mucosal injury. CAD treatment substantially attenuated these elevations, particularly at higher dosages. Additionally, CAD markedly reduced oxidative stress in intestinal tissues, as shown by normalized antioxidant levels and decreased reactive oxygen species. Histological analysis supported these findings, showing better-preserved villi structures in CAD-treated groups. Furthermore, CAD significantly reduced IL-6 and IL-8 expression post-LPS stimulation and effectively regulated the NLRP3 inflammasome pathway, decreasing associated factors like NLRP3, Caspase-1, IL-1b, and IL-18.

**Discussion:**

The study demonstrates CAD's therapeutic potential in alleviating LPS-induced intestinal injuries. The protective effects are primarily attributed to its anti-inflammatory and antioxidative actions and modulation of the NLRP3 inflammasome pathway.

## Graphical Abstract

Cecropin AD (CAD) mitigates LPS-induced intestinal damage in chickens, reducing oxidative stress and modulating inflammatory markers through the NLRP3 inflammasome pathway.

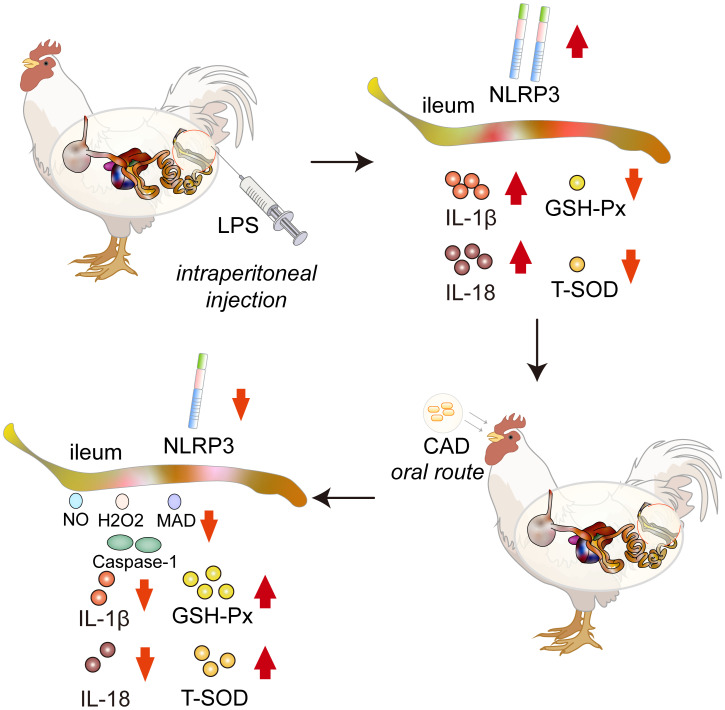

## Introduction

The intestinal tract is pivotal in digestion, detoxification, and nutrient absorption, underpinning overall health (1, 2). Lipopolysaccharide (LPS), a key component of Gram-negative bacterial cell walls (3), has attracted significant attention due to its harmful impact on the intestinal environment. LPS is comprised of lipid A, a core polysaccharide, and O antigen, with lipid A identified as the main toxic element of LPS (4). LPS engages with Toll-like receptor 4 (TLR4) (5), triggering pro-inflammatory cascades and ensuing widespread inflammation (6). Notably, LPS is a primary activator of TLR4 signaling, linked to the development of various inflammatory diseases and cancers (6). LPS-induced changes in the intestinal mucosa can hinder nutrient uptake, and in extreme cases, lead to systemic inflammation (7, 8). In China’s poultry industry, chickens are frequently exposed to LPS-rich environments (9, 10), rendering them prone to its associated immune challenges (11, 12). This exposure risks their overall health and productivity, making the exploration of LPS’s effects and counteractive measures a priority for enhancing avian welfare and industry yield (13).

Reactive Oxygen Species (ROS), found across various cellular contexts, play roles in numerous physiological and pathological processes (14). Interestingly, ROS exhibit a robust association with TLR4 and facilitate the release of IL-1β and IL-18 via the NLRP3 inflammasome activation. While modest ROS concentrations are indispensable for cellular operations (15, 16), excessive ROS levels, overwhelming the body’s antioxidant capacity, result in oxidative stress and cellular damage (17, 18). In inflamed conditions, increased ROS levels enhance inflammatory pathways, an effect amplified by LPS (19). The oxidative damage from LPS relates to shifts in free radicals, antioxidative enzyme behaviors, and changes in oxidative byproducts (20, 21). Notably, LPS exposure precipitates a surge in malondialdehyde (MDA) content in chicken intestinal mucosa while reducing the activities of SOD and GSH-Px (22). In conclusion, ROS are central to LPS-induced intestinal damage, highlighting the need for a thorough understanding of their role to prevent and treat intestinal injuries effectively.

The innate immune system of the intestinal tract acts as the primary defense against external threats (23). At the heart of this defense is the NLRP3 inflammasome of the NOD-like receptor family, integral to inflammatory responses (24). The NLRP3 inflammasome comprises the apoptosis-associated speck-like protein (ASC), NLRP3 protein, and Caspase-1. Notably, Caspase-1 plays a pivotal role in maturing and releasing IL-1β and IL-18 (25, 26). Additionally, the NF-κB protein, functioning as the inflammasome’s upstream signal, can activate the NLRP3 protein (27).NLRP3 inflammasome activation unfolds in two stages (28): initially, cytokines and bacterial elements provide priming signals that influence the expression patterns of NLRP3 and pro-IL-1β. This phase is followed by various stimuli like potassium efflux, ROS production, or cellular infections that further stimulate NLRP3 (29). Activation mechanisms can be divided into classical and non-classical pathways (30). The classical method involves a two-step signaling process: an initial phase where the TLR4 pathway activation leads to NF-κB activation, subsequently activating precursor proteins such as IL-18 (31–33). Then, the inclusion of ASC supports the assembly of the NLRP3, ASC, and pro-Caspase-1 combination, leading to the release of IL-1β and IL-18 and triggering inflammation. In contrast, the non-classical pathway, driven by Caspase-11 and primarily activated by LPS, also promotes inflammatory responses (34). It’s important to note that improper activation of the NLRP3 inflammasome is linked to various conditions, ranging from Inflammatory Bowel Disease (IBD) (35) to acute myocardial infarctions (36). In the face of LPS aggression, the precise modulation of NLRP3 inflammasome activity becomes paramount to maintaining intestinal integrity.

Herein lies the potential of antimicrobial peptides such as CAD, which has demonstrated broad-spectrum antimicrobial efficacy (37, 38). Specifically, lab-produced CAD exhibits enhanced antimicrobial activity (39) and when encased in microgels, protects against hydrolysis by proteases and also reduced peptide-mediated cytotoxicity (40). CAD’s interaction with cell membranes and intracellular targets presents a novel angle in the quest for immunomodulatory and protective agents in livestock (41, 42). Cecropin’s application across various livestock sectors—from pigs (43) and poultry (44) to ruminants (45) and aquatic species (46)—underscores its diverse benefits, including growth promotion, immune enhancement, and disease prevention. Its application across diverse animal sectors points to its promise as a non-antibiotic, eco-friendly feed additive. However, the specific impact of CAD on LPS-induced intestinal challenges in chickens, particularly in the modulation of the NLRP3 inflammasome, remains uncharted territory.

Given the complex interplay of the NLRP3 inflammasome in immune responses and the potential of CAD in immune regulation, this study embarks on an innovative investigation into CAD’s role in protecting against LPS-induced intestinal mucosal injury in chickens. We closely examine the dynamics of the NLRP3 inflammasome within the chickens intestinal environment and explore how CAD affects its expression and activity. Our goal is to illuminate the protective mechanisms of CAD and to contribute to the development of new strategies for maintaining intestinal health in poultry.

## Materials and methods

### Ethics approval and consent to participate

All animal work in this study met the minimum standards of animal welfare as described in the International Guiding Principles for Biomedical Research involving Animals (at https://grants.nih.gov/grants/olaw/Guiding_Principles_2012.pdf). The handling of birds was performed in accordance with the Guidelines of the Animal Care and Use Committee and approved by the Institute of Animal Husbandry and Veterinary Medicine (Permit number: BUA2022070). All efforts were made to alleviate animal suffering and to improve their quality of life.

### Birds, management, and experimental design

Sixty 14-day-old SPF-grade chicks were sourced from Beijing Boehringer Ingelheim Vetsuisse Ltd. The Cecropin AD (Q/DXN 063-2021) was obtained from Viteling Antibiotic-Free Breeding Technology (China). The LPS used was Lipopolysaccharides from Escherichia coli O55:B5, γ-irradiated and designated as BioXtra, suitable for cell culture use (Shanghai Yuan Ye Biotechnology Co., Ltd., EC Number 297-473-0, MDL number MFCD00164401, China). Following a one-week acclimatization period, chicks were randomly divided into six groups: a control group, a model group, three CAD dosage groups (40, 80, and 160 mg/mL), and a drug control group (160 mg/mL). Each group, consisting of 10 chicks, was housed separately in individual units within an SPF aviary until the experiment’s end. Chicks had uninterrupted access to feed and water. For the LPS stress model, chicks in the control and drug control groups received 7.5 ml/kg of physiological saline at the study’s outset. In contrast, the other groups were administered the same dose of LPS at 35 days of age through intraperitoneal injection. The CAD dosages were meticulously selected based on our preliminary *in vitro* antibacterial assays, where CAD exhibited Minimum Inhibitory Concentrations (MICs) of 20 µg/mL and 40 µg/mL against Escherichia coli (ATCC 25922) and Staphylococcus aureus (ATCC 25923), respectively. We calculated the *in vivo* dosages as 2x, 4x, and 8x the average of these MICs, translating to 60 mg/kg, 120 mg/kg, and 240 mg/kg for the low, medium, and high dosage groups. Over a 14-day period, CAD was administered orally at a volume of 1ml per dose. Considering the average weight of the chicks during this period was approximately 0.67kg, the corresponding CAD concentrations for the dosage groups were determined as 40 mg/mL, 80 mg/mL, and 160 mg/mL. After injections, close monitoring was conducted on the chicks’ physiological and behavioral aspects, including alertness, weight, feed intake, respiration, and movement.

### Sample collection and procedure

Twelve hours after the intraperitoneal injection of the LPS solution, three chicks from each group were randomly chosen, totaling 18 chicks. Blood samples were carefully drawn through cardiac puncture and immediately placed into anticoagulant tubes to prepare plasma samples. Following this, and in strict compliance with ethical standards for animal research, the chosen chicks were elthanized with inhaled carbon dioxide gas. Afterward, ileum sections were carefully extracted, rinsed with physiological saline, and specific segments were fixed in a 10% formalin solution for future histological analysis. Remaining ileal tissue samples were stored at -80°C. These samples will undergo thorough analyses, including DAO and D-LA activity, oxidative stress indicators, and antioxidative capabilities. Additionally, they will be used for total RNA and protein extractions for subsequent experiments.

### Assessment of plasma intestinal barrier biomarkers and antioxidant status

The activity and concentration of serum DAO (MA60560) and D-LA (MA60570, Zhonghao Biotechnology Co., Ltd., Beijing, China) were determined using ELISA kits. Concurrently, biochemical activities of antioxidants and oxidative stress markers, such as T-SOD, GSH, GSH-PX, H_2_O_2_, NO, and MDA in the intestinal mucosa supernatants and plasma, were measured with commercial biochemistry kits following the manufacturer’s guidelines (Nanjing Jiancheng Bioengineering Institute, Nanjing, China). Notably, metrics from tissue homogenates were expressed in units per milligram of protein.

### Biochemical analysis of the concentration of cytokines in the intestinal mucosa

The expression profiles of pivotal cytokines, encompassing IL-1β (H002-1-1) and IL-18 (H015-1-1) were rigorously quantified in the supernatants extracted from the intestinal mucosa. For this endeavor, we employed commercially available biochemistry kits, strictly adhering to the procedural guidelines specified by the manufacturer (Nanjing Jiancheng Bioengineering Institute, Nanjing, China).

### Analysis of intestinal histomorphology

Intestinal tissue samples were preserved in 4% paraformaldehyde for 24 hours. After fixation, samples followed standard paraffin embedding procedures. Sections, 4µm thick, were stained with hematoxylin and eosin (H&E) to enable morphological visualization. These sections were examined under a DP80 Digital light microscope (Olympus, Tokyo, Japan), and relevant images were captured. Parameters such as villus height (from the villus tip to the crypt base) and crypt depth (from the invagination opening to the base above the lamina muscularis mucosae) were assessed using the ImagePro Plus 6.0 software (Media Cybernetics). At least ten distinct views from each sample were chosen for observation to ensure the accuracy of the experiment.

### RNA extraction and real-time quantitative PCR

Total RNA was extracted from mucosal scrapings of intestinal tissue using the TRIzol reagent (Takara, Dalian, China) as per the manufacturer’s instructions (47). The RNA’s concentration and purity were determined with a Nanodrop 2000C (Thermo Fisher Scientific, Waltham, MA, USA), ensuring an A260/280 absorbance ratio. The PrimeScript RT Reagent Kit (Takara, Dalian, China) was employed to synthesize cDNA. Quantitative PCR (qPCR) analyses were conducted on the CFX96 Real-Time System (Bio-Rad, Hercules, CA, USA) with conditions set as: 95°C for 2 minutes for initial denaturation, followed by 40 cycles of 95°C for 10 seconds and the annealing/extension temperature for 30 seconds, and fluorescence signal collection. (as listed in **Table 1**) The melt curve analysis ranged from 65~95°C. Amplification efficiencies for target genes were between 95% and 105%. Each qPCR reaction consisted of 10 µL TB GreenTM Premix (Takara), 0.4 µL of both forward and reverse primers, 1.5 µL cDNA, and 7.7 µL DNase/RNase-Free Deionized Water (Tiangen, Beijing, China). All samples were analyzed in triplicate, with cycle threshold (Ct) values normalized to β-actin expression levels. Relative mRNA levels were determined using the 2-△△Ct method (48).

**Table 1:** Primers used for quantitative real-time polymerase chain reaction (qRT-PCR).

Genes	Sequence (5’-3’)	Product Length (bp)	Accession Number
**Claudin 1**	F:GACCAGGTGAAGAAGATGCGGATG R:CGAGCCACTCTGTTGCCATACC	107 bp	NM_001013611.2
**Occludin**	F:TCATCGCCTCCATCGTCTAC R:TCTTACTGCGCGTCTTCTGG	240 bp	XM_025144248.1
**Zona Occludens 1 (ZO-1)**	F:CTTCAGGTGTTTCTCTTCCTCCTC R:CTGTGGTTTCATGGCTGGATC	131 bp	XM_015278981.2
**Mucin 2**	F:CCCTCACCCAGCCCGACTTC R:GCCGTTGGTGGAGGTGTTACAG	179 bp	JX284122.1
**GAPDH**	F:CCCCCATGTTTGTGATGGGT R:GCACGATGCATTGCTGACAA	74 bp	NM_204305.2
**IL-1β**	F:GCCTGCAGAAGAAGCCTCG R:GGAAGGTGACGGGCTCAAAA	210 bp	NM_204524.2
**IL-18**	F:GAGCCCGTTCGGGGGA R:GCATCGCATTCCAGCTCATC	171 bp	XM_015297948.4
**IL-6**	F:CTCGTCCGGAACAACCTCAA R:TCAGGCATTTCTCCTCGTCG	85 bp	NM_204628.2
**IL-8**	F:TGCTCTGTCGCAAGGTAGGA R:GGTCCAAGCACACCTCTCTT	188 bp	NM_205498.2
**Caspase-1**	F:GCCTGAATCAGCACCGTAGT R:AGGGCTGACAGTATTCCTGC	179 bp	XM_011311252.1
**NLRP3**	F:CCTGAGTGACACCGAGCTG R:TGTAGAAGTGCTCAGCCCCA	100 bp	NM_001348947.2

### Western blot analysis of protein expression

Intestinal tissue samples, each weighing approximately 40 mg, were lysed using RIPA protein lysis buffer. Tissue homogenization was performed for 1 minute, repeated twice, and the homogenate was incubated at 4°C for 30 minutes. The lysate was then centrifuged at 12,000 rpm at 4°C for 5 minutes, with the supernatant being carefully transferred to a fresh centrifuge tube and stored at -80°C. Protein concentrations were ascertained using the BCA assay, with absorbance readings taken at 562 nm. By establishing a standard curve as per manufacturer guidelines, protein content within the samples was deduced.

Subsequent to protein quantification, proteins were separated using 15% SDS-PAGE and transferred onto membranes at a constant voltage of 100V for 120 minutes. Following transfer, membranes underwent a 2-hour blocking step. After a thorough wash in TBST, membranes were probed with primary antibodies targeting NLRP3 (CST # 15101, Cell Signaling Technology) and GADPH (CST # 2118, Cell Signaling Technology), undergoing an overnight incubation at 4°C with gentle agitation. Post-primary incubation, membranes were treated with appropriately diluted secondary antibodies and incubated at room temperature for 90 minutes with gentle shaking. ECL detection reagent was then applied to the membranes for chemiluminescent detection. Band intensities were captured, and quantitative analyses were conducted using Image J software.

### Statistical analysis

Data were analyzed using one-way analysis of variance (ANOVA) with GraphPad Prism version 8.3 (GraphPad Software Inc., San Diego, CA) and SPSS 20 (SPSS Inc., Chicago, IL, USA). Experimental results are presented as mean ± standard deviation (SD). Tukey’s *post hoc* test was applied for pairwise comparisons among treatment groups. The equation △Ct = mean value of target gene − mean value of internal reference gene was utilized, with △△Ct = △Ct − mean value of control group. A p-value less than 0.05 was deemed statistically significant.

## Results

### Cecropin AD alleviates LPS-induced intestinal mucosal injury

**Figure 1:**
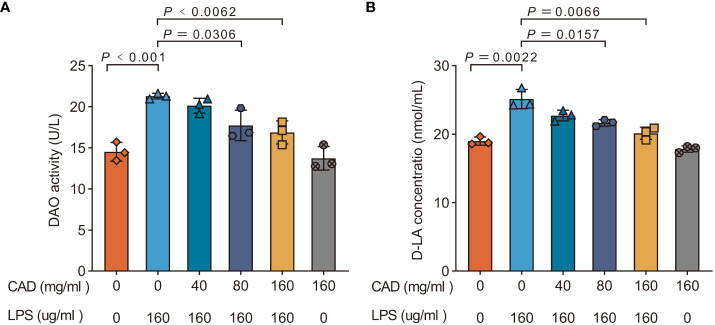
Effects of CAD on the DAO and D-LA activities of plasma in chickens. **(A)** DAO; **(B)** D-LA. Experiments were performed as three biologically independent experiments, and the mean ± S.D. (n = 3) were shown. *P* values were determined by non-parametric one- way ANOVA.

As shown in **Figure 1**, the LPS group exhibited a significant increase in DAO and D-LA activity compared to the control group (*P*<0.001). Administration of medium and high doses of CAD effectively attenuated the LPS-induced rise in DAO and D-LA levels. Specifically, high-dosage CAD resulted in a marked reduction in DAO and D-LA activity compared to the LPS group (*P*<0.01), with a similar trend observed for the medium dosage (*P*<0.05). The DAO and D-LA levels in the high-dosage CAD control group remained comparable to the control, indicating that CAD, even at high concentrations, doesn’t harm the intestinal mucosa. In essence, CAD effectively counteracts the LPS-induced intestinal mucosal injuries in chickens.

### Cecropin AD influences mRNA expression of tight junction proteins and barrier function biomarkers in chicken intestinal mucosa

**Figure 2:**
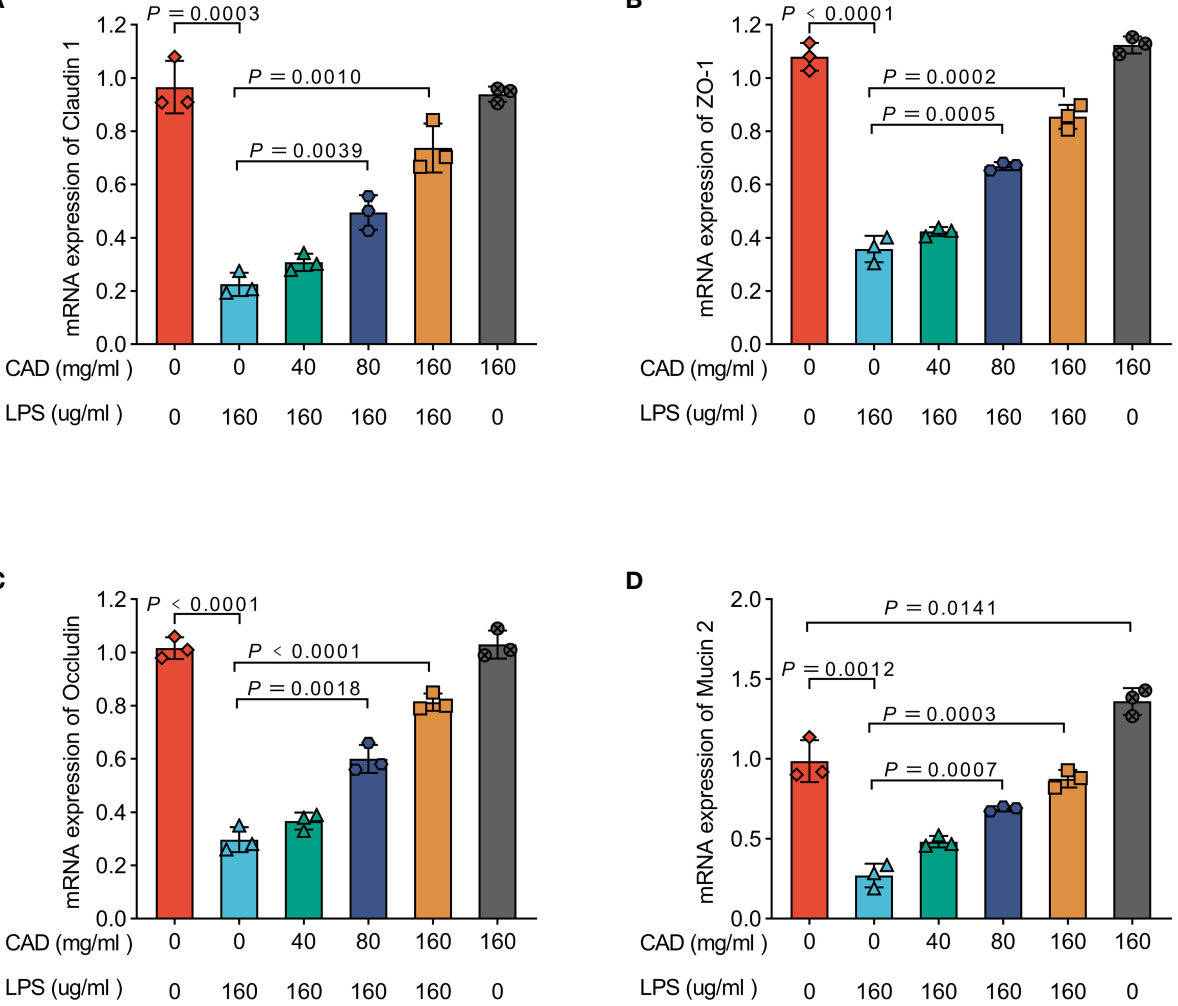
Effectsof CAD on Claudin 1, ZO-1, Occludin, and Mucin 2 mRNA Levels in Chicken Intestinal Mucosa. **(A)** Claudin 1; **(B)** ZO-1; **(C)** Occludin. **(D)** Mucin 2. Experiments were performed as three biologically independent experiments, and the mean ± S.D. (n = 3) were shown. *P* values were determined by non-parametric one- way ANOVA.

As illustrated in **Figure 2**, the mRNA levels of Claudin 1, Occludin, Zona Occludens 1 (ZO-1), and Mucin 2 in the LPS group were significantly reduced compared to the control group (*P*<0.005). The administration of medium and high doses of CAD effectively mitigated this reduction induced by LPS (*P*<0.005). A dose-dependent trend was observed across low, medium, and high dosages. Notably, in the high-dosage CAD control group, the levels of Claudin 1, Occludin, and Zona Occludens 1 (ZO-1) were comparable to those in the control group, indicating that high-dose CAD did not adversely affect these parameters. However, under high-dose CAD treatment, Mucin 2 levels were significantly elevated compared to the control group (*P*<0.05), suggesting a specific upregulation of this marker.

### Cecropin AD mitigates LPS-induced ROS generation and oxidative stress in intestinal tissues

**Figure 3:**
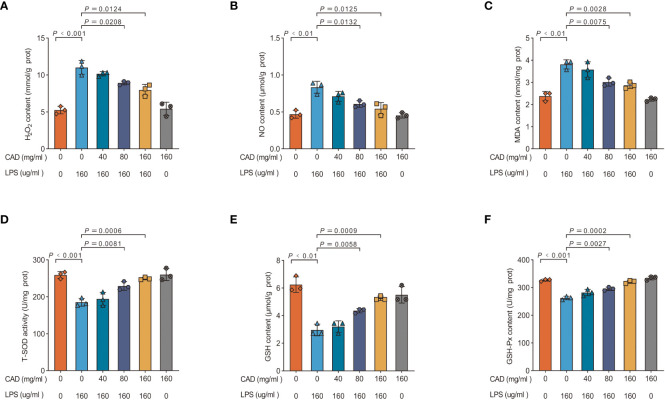
Effects of CAD and LPS antioxidant enzymes and MDA levels. **(A)** H_2_O_2_ content; **(B)** NO content; **(C)** MDA content; **(D)** T-SOD activity; **(E)** GSH content; and **(F)** GSH-Px activity of ileum in chickens. Experiments were performed as three biologically independent experiments, and the mean ± S.D. (n = 3) were shown. *P* values were determined by non-parametric one- way ANOVA.

This study assessed the ability of CAD to counteract LPS-induced oxidative stress and the production of ROS-like molecules in chicken intestinal tissues. The evaluation encompassed six markers: H_2_O_2_, NO, MDA concentrations, and T-SOD, GSH, and GSH-Px activities (**Figure 3**). The LPS-treated group showed significantly elevated levels of H_2_O_2_, MDA, and NO (*P*<0.01) and reduced activities of T-SOD, GSH (*P*<0.01), and GSH-Px (*P*<0.001) when compared to controls. Medium and high-dosage CAD treatments significantly decreased H_2_O_2,_ NO and MDA concentrations (*P*<0.05) and enhanced T-SOD activities (*P*<0.01). Additionally, high-dose CAD treatment significantly elevated GSH levels (*P*<0.0001) and restored GSH-Px activity (*P*<0.001). Notably, the low-dosage CAD group did not show significant alterations in these indicators. Collectively, these results indicate that CAD, especially at medium and high doses, effectively counters ROS-like molecule production and alleviates oxidative stress in LPS-challenged intestinal tissues.

### Histopathological observations of intestinal tissues

**Figure 4:**
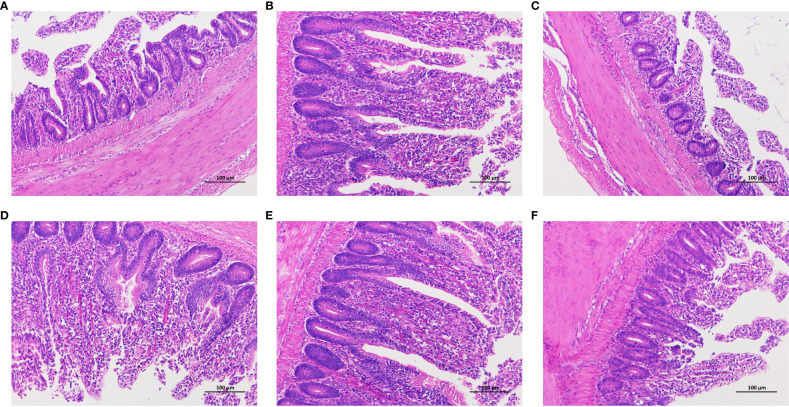
Effect of CAD and LPS on intestinal inflammatory in chickens (200x). **(A)** control group; **(B)** LPS-treated group; **(C)** LPS-treated with 40 mg/mL CAD group; **(D)** LPS-treated with 80 mg/mL CAD group; **(E)** LPS-treated with 160 mg/mL CAD group; and **(F)** 160 mg/mL CAD group.

The histological analysis of chicken intestinal tissues post-Hematoxylin and Eosin (HE) staining is shown in **Figure 4**. The control group samples exhibited clear and well-preserved villi structures. In contrast, the tissues treated with LPS revealed epithelial cell dislodgment from mucosal glands and significant lymphocyte infiltration. With the increase in CAD dosage, the clarity and integrity of the villi structures were enhanced, particularly at dosages of 80 mg/mL and 160 mg/mL, where the protective effects were clearly evident. Notably, at these dosages (**Figures 4D, E**), the submucosal and muscular layers appeared normal, with reduced lymphocyte infiltration and no other anomalies. The high-dosage CAD group resembled the control group, maintaining normal intestinal architecture. This underscores the potential of CAD in alleviating LPS-induced intestinal damage in chickens.

### Cecropin AD mitigates LPS-triggered inflammatory reactions in intestinal tissues

**Figure 5:**
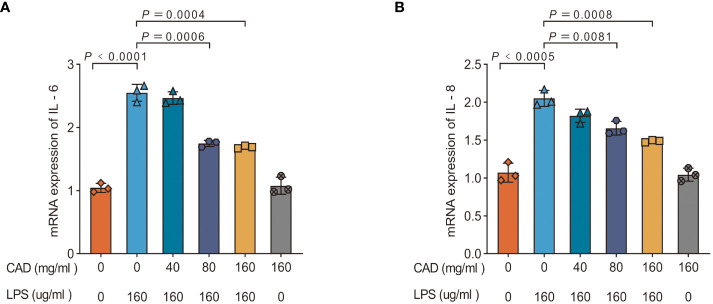
Effect of CAD and LPS on the mRNA expressions of pro-inflammatory cytokine-related genes in intestinal tissues in chickens. **(A)** IL-6; **(B)** IL-8. Experiments were performed as three biologically independent experiments, and the mean ± S.D. (n = 3) were shown. *P* values were determined by non-parametric one- way ANOVA.

To elucidate CAD’s efficacy in counteracting LPS-induced inflammatory responses, we assessed mRNA levels of IL-6 and IL-8 in chicken intestinal tissues. **Figure 5** reveals a pronounced upregulation of IL-6 and IL-8 mRNA expression in the LPS group compared to the control (*P*<0.0005). Relative to the model group, a significant reduction in these mRNA levels was observed in the medium-dose CAD group (*P*<0.01). The high-dose CAD cohort evidenced an even more pronounced decline (*P*<0.001), while the drug control group remained unaffected, indicating the high dosage’s non-inflammatory nature. This accentuates CAD’s potential in dampening LPS-induced inflammatory cascades in intestinal tissues.

### Cecropin AD suppresses NLRP3 inflammasome expression and activation in intestinal tissues

**Figure 6:**
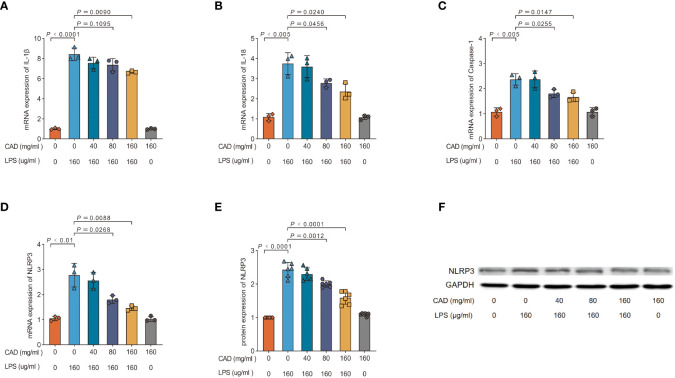
Effect of CAD and LPS on NLRP3 inflammasome expression and activation in intestinal tissues in chickens. **(A)** IL-1β; **(B)** IL-18; **(C)** Caspase-1; **(D)** NLRP3; **(E, F)** NLRP3 protein. Experiments were performed as three or six biologically independent experiments, and the mean ± S.D. (n = 3 or 6) were shown. *P* values were determined by non-parametric one- way ANOVA.

Our investigation delved into CAD’s modulatory impact on the NLRP3 inflammasome by probing the expression of NLRP3, Caspase-1, IL-1β, IL-18 mRNA, and NLRP3 protein. As depicted in **Figures 6A–F**, the LPS group exhibited a pronounced upswing in NLRP3, Caspase-1, IL-1β, IL-18 mRNA, and NLRP3 protein levels relative to the control (*P*<0.01). Both medium and high CAD concentrations curtailed the NLRP3 inflammasome’s expression and activation. Specifically, against the LPS benchmark, the high-dose CAD regimen revealed a significant downtick in NLRP3 and IL-1β mRNA expression (*P*<0.01) and appreciable reductions in Caspase-1 and IL-18 mRNA levels (*P*<0.05). Additionally, NLRP3 protein levels in both the medium and high CAD cohorts were substantially diminished (*P*<0.005). In contrast, low-dose CAD administration yielded no discernible shifts in these markers. These insights underscore CAD’s anti-inflammatory prowess via NLRP3 inflammasome modulation.

### Cecropin AD modulates IL-1β and IL-18 concentrations in intestinal tissues

**Figure 7:**
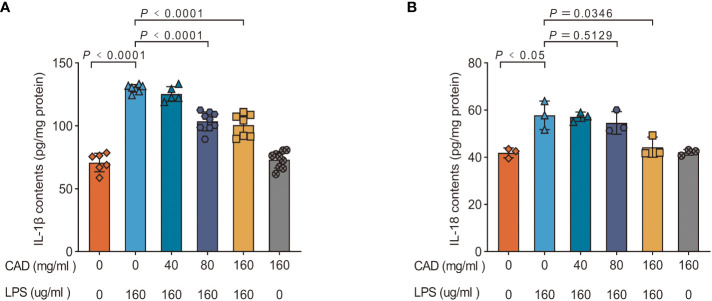
Effect of CAD and LPS on the IL-1β and IL-18 contents in intestinal tissues in chickens. **(A)** IL-1β; **(B)** IL-18. Experiments were performed as three or nine biologically independent experiments, and the mean ± S.D. (n = 3 or 9) were shown. *P* values were determined by non-parametric one- way ANOVA.

Our investigation further extended to quantifying the concentrations of IL-1β and IL-18 within chicken intestinal tissues, with findings detailed in **Figure 7**. Compared to the control cohort, the LPS-treated group manifested significantly elevated levels of IL-1β and IL-18 (*P*<0.05). Intriguingly, both the medium and high dosages of CAD induced a substantial decrement in IL-1β concentrations within the intestinal samples (*P*<0.01). Conversely, the low-dosage CAD group showcased no discernible alterations in IL-18 and IL-1β levels. These outcomes insinuate that CAD’s modulatory effects on IL-1β and IL-18 production are potentially tethered to its interactions with the NLRP3 inflammasome.

## Discussion

In our investigation, Diamine oxidase (DAO) and D-Lactate (D-LA) served as primary biomarkers for assessing gut permeability and mucosal integrity (49, 50). Their escalated plasma levels post-LPS exposure indicated a compromise in mucosal integrity, typically resulting from pathogenic assaults (51). Our findings corroborate this with the established chicken intestinal damage model using an LPS concentration of 160 μg/ml. However, beyond DAO and D-LA, we also observed significant alterations in the mRNA levels of tight junction proteins and barrier function biomarkers such as Claudin 1, Occludin, ZO-1, and Mucin 2. The reduction in these markers in the LPS group further emphasized the extent of intestinal barrier disruption. Remarkably, CAD administration, particularly at medium and high doses, not only mitigated the DAO and D-LA levels but also positively influenced the expression of these crucial tight junction proteins, aligning with the restoration of gut integrity. The parallel restoration of Claudin 1, Occludin, and ZO-1 levels, alongside the notable increase in Mucin 2 in response to higher CAD doses, provides additional insights into the multifaceted protective mechanisms of CAD against intestinal mucosal damage.

In the intestinal environment, non-enzymatic antioxidants such as glutathione (GSH) and the thioredoxin system are instrumental in sustaining cellular redox equilibrium (52, 53). Key antioxidants dependent on GSH, including glutathione peroxidase (GSH-Px), superoxide dismutase (SOD), and glutathione reductase (GR), serve as critical bulwarks against oxidative stress. SOD, in particular, counters reactive oxygen species (ROS), a principal factor in oxidative stress responses (54). Among ROS, hydrogen peroxide (H_2_O_2_) is efficiently neutralized into water by enzymes like glutathione peroxidase and catalase. GSH-Px, moreover, plays a dual role: reducing H_2_O_2_ and lipid peroxides, thereby shielding against ROS and pathogen-induced non-pathogenic inflammatory responses (55). Malondialdehyde (MDA), an indicator of oxidative stress, emerges as a result of intracellular lipid peroxidation (56). Our findings indicate that LPS stimulation escalates H_2_O_2_, NO, and MDA levels, triggering oxidative stress in intestinal epithelial cells. This stress is characterized by a reduction in GSH, GSH-Px, and total SOD (T-SOD) activities. However, CAD administration markedly alleviated this oxidative stress and ROS-mediated damage. This ameliorative impact is reflected in the diminished concentrations of H_2_O_2_, NO, and MDA, and in the revitalized activities of T-SOD and GSH-Px, effectively restoring the GSH pool.

IL-6, akin to C-reactive protein, is gaining prominence as a biomarker in monitoring inflammatory responses, particularly in cases of cancer, infections, or autoimmune disorders (57). The pivotal role of IL-6 in driving inflammatory processes accounts for its burgeoning significance in clinical diagnostics. Furthermore, cells bearing Toll-like receptors (TLRs) — notably macrophages and smooth muscle cells — are capable of producing and releasing IL-8 (58). Endothelial cells, too, contribute to this cytokine milieu, storing IL-8 within specialized Weibel-Palade bodies (59). Consistent with these cellular mechanisms, our findings reveal an upregulation in IL-6 and IL-8 expression following LPS induction, highlighting the inflammation’s systemic nature. This leads us to consider the broader implications of CAD as a therapeutic agent. Demonstrating both anti-inflammatory and antioxidative effects, CAD emerges from our research as a robust candidate for managing conditions characterized by escalated oxidative stress and inflammatory responses. Its efficacy in modulating key inflammatory biomarkers like IL-6 and IL-8 not only validates its therapeutic potential but also marks it as a promising alternative in the realm of anti-inflammatory interventions.

Research into the dual roles of NLRP3 inflammasomes in autoinflammatory syndromes has been a focal point for over a decade, yet their exact role in conditions like inflammatory bowel diseases remains elusive (60). Studies indicate that NLRP3 inflammasomes can both enhance immune tolerance and epithelial barrier integrity (61, 62), while their overactivation may disrupt intestinal immune homeostasis (63). This complexity underscores the significance of our research in delineating the multifaceted nature of NLRP3 inflammasomes and the impact of interventions like CAD. Our findings reveal that LPS stimulation activates NLRP3 inflammasomes, leading to increased cytokine production, such as IL-1β and IL-18. Although such immune responses may be beneficial under certain conditions, unchecked activation can be detrimental. Notably, our study demonstrates that CAD effectively modulates this response, as evidenced by the decreased expression of NLRP3, Caspase-1, IL-1β, and IL-18 following CAD treatment. This suggests CAD’s therapeutic potential, especially considering its safety profile. Moreover, CAD’s efficacy in reducing DAO activity, a benchmark for intestinal mucosal damage, further indicates its role in mitigating intestinal inflammation and injury. The determination of an optimal CAD dosage is pivotal; our data suggests that while low doses are less effective, medium to high doses offer more pronounced benefits. In the broader context, assessing CAD against other therapeutic agents targeting similar inflammatory pathways is essential. This comparison is vital to position CAD within the therapeutic landscape, highlighting its unique advantages and potential limitations. While CAD shows promise over certain treatments like non-steroidal anti-inflammatory drugs, methotrexate, heparin, and hormone therapy, in terms of side effects, cost, and ease of administration (64–67), other well-established therapies might confer additional benefits beyond managing inflammation and oxidative stress (68, 69).

While our research establishes CAD as an effective agent in an LPS-induced chicken model for intestinal damage, extrapolating these results to other species, particularly humans, invites further scrutiny. Critical factors such as the long-term impact of CAD, potential toxicities at higher doses, and its interactions with existing medications are pivotal considerations for its integration into broader therapeutic protocols. This underscores the need for extensive research and comparative studies to elucidate CAD’s comprehensive therapeutic profile, its underlying mechanisms, and potential constraints in managing inflammation. Additionally, the gastrointestinal tract’s role in systemic homeostasis, especially in relation to factors like malnutrition and impaired mucosal barrier function contributing to diseases such as enteritis, underlines the importance of regulatory components like NLRP3 inflammasomes. These inflammasomes, influencing key innate immune cells such as monocytes, macrophages, and dendritic cells, play a vital role in maintaining intestinal equilibrium and are implicated in the etiology of inflammatory bowel diseases (70, 71). It is also essential to acknowledge the limitations of the LPS-induced model which, despite providing a controlled setting, may not fully mimic the intricate biological, environmental, and nutritional interplays encountered in natural conditions. This gap necessitates comprehensive research to validate our findings’ practical relevance in chicken intestinal health management. In sum, our study highlights CAD’s potential in alleviating LPS-induced intestinal damage, evidenced by reduced DAO and D-LA levels. Nevertheless, a thorough understanding of the treatment mechanisms and their applicability in real-world scenarios remains a subject for future exploration. Our findings contribute significantly to the growing body of knowledge in intestinal health management, paving the way for innovative therapeutic approaches to counter mucosal damage.

Future research initiatives, building on our current findings, should be directed towards decoding the intricate mechanisms by which CAD modulates the activity of NLRP3 inflammasomes. Furthermore, exploring the combined use of CAD with other therapeutic agents holds the promise of discovering synergistic treatment modalities. Such collaborative therapeutic strategies could significantly enhance the efficacy of treatments, especially for complex conditions like inflammatory bowel disease. This research trajectory not only broadens our comprehension of CAD’s function in inflammation but also heralds the potential for developing more precise and potent interventions for various inflammatory disorders. The pursuit of this knowledge is crucial for advancing the field of inflammation management and for the formulation of targeted therapeutic strategies.

## Conclusions

In conclusion, our study elucidates the pivotal role of CAD in mitigating LPS-induced inflammatory responses, ameliorating intestinal lesions in chickens, reducing oxidative stress, and attenuating the expression and activation of NLRP3 inflammasomes. These findings underscore CAD’s crucial contribution to preserving intestinal function’s stability and integrity. The research not only showcases CAD’s efficacy as a therapeutic agent but also underscores its potential as a preventive measure against intestinal inflammation. Our study thereby offers significant insights into CAD’s utility in the realm of intestinal health management, paving the way for its potential application in broader therapeutic contexts.

## Data availability statement

The raw data supporting the conclusions of this article will be made available by the authors, without undue reservation.

## Ethics statement

The animal study was approved by Institute of Animal Husbandry and Veterinary Medicine. The study was conducted in accordance with the local legislation and institutional requirements.

## Author contributions

YZ: Data curation, Formal analysis, Investigation, Methodology, Validation, Visualization, Writing – original draft, Writing – review & editing. TL: Data curation, Investigation, Validation, Writing – original draft. YL: Investigation, Writing – review & editing. TZ: Investigation, Writing – review & editing. MD: Investigation, Methodology, Writing – review & editing. QZ: Investigation, Methodology, Writing – review & editing. XW: Investigation, Writing – review & editing. GH: Conceptualization, Funding acquisition, Methodology, Project administration, Resources, Supervision, Writing – review & editing.
